# Influence on the patient’s oral hygiene depending on the treatment performed by either one or different pre-graduate practitioners — a randomized, controlled, clinical short-term trial

**DOI:** 10.1007/s00784-022-04501-1

**Published:** 2022-04-29

**Authors:** Marco M. Herz, Nora Celebi, Thomas Bruckner, Valentin Bartha

**Affiliations:** 1 Department of Conservative Dentistry and Periodontology, University Hospital of Tuebingen, Osianderstr. 2-8, 72076 Tuebingen, Germany; 2 PHV-Dialysezentrum Waiblingen, Beinsteiner Str. 8/3, 71334 Waiblingen, Germany; 3 Institute of Medical Biometry, Faculty of Medicine, University of Heidelberg, Im Neuenheimer Feld 130.3, 69120 Heidelberg, Germany; 4 Department of Conservative Dentistry, University Hospital of Heidelberg, Im Neuenheimer Feld 400, 69120 Heidelberg, Germany

**Keywords:** Oral hygiene, Plaque control, Gingival bleeding, Periodontitis, Dental students

## Abstract

**Objectives:**

Plaque control by improved domestic oral hygiene is essential in periodontal treatment. However, changing treatment providers may interfere with building a dentist-patient relationship and in turn affect treatment success. The aim of this randomized, controlled, prospective short-term study was to determine the influence of either one or four different pre-graduate practitioners on patients’ oral hygiene parameters during active periodontal therapy.

**Material and Methods:**

A total of 55 patients with periodontitis were allocated to two groups. Within the group “continuous treatment” (CT, *n* = 27), each patient was treated by one individual practitioner over the treatment period. For patients of the group “discontinuous treatment” (DT, *n* = 28), treatment in each session was performed by a different practitioner. Periodontal parameters (BOP, PBI, and PCR) were assessed at two timepoints: T1 (baseline) and T2 (end of active therapy).

**Results:**

With CT, the PBI improved in 93% of the patients, compared to 71% with DT (*p* = 0.048). T1-T2 intragroup analysis showed a statistically significant improvement of all observed clinical parameters with no differences in ∆PBI, ∆BOP, and ∆PCR. Spearman’s correlation analysis revealed a weak correlation between PCR and BOP of CT only.

**Conclusions:**

In the present study, improvement of all parameters was comparable between the groups. PBI, as a parameter displaying patient’s domestic plaque control compliance, improved in more patients from CT than DT. This is possibly indicating an advantage of continuous treatment by one single practitioner.

**Clinical relevance:**

Treatment by either a single practitioner or by multiple, constantly changing practitioners might influence patients’ compliance to modify their behaviour when medically necessary.

## Introduction

Today, the so-called dental medical care centres spread at the expense of the traditional practice model. In March 2020, there were already over 1000 care centres, with around 76% of them being located in high-income urban areas [1]. Many of these new centres typically employ several dentists treating patients often randomly, yet staff fluctuation in these centres is quite high [2]. As a result, treatment of patients by different practitioners at each appointment is nothing unusual.

However, there is some concern that changing treatment providers may interfere with building a dentist-patient relationship and in turn affect treatment success and patient compliance [3]. The situation is similar at university dental schools. It is common that one patient is treated by multiple students [3, 4]. At university, however, this concept has several advantages: First, the students gain much more experience by treating and examining an increased number of patients. Furthermore, more students have more treatment times available than one individual student, which in turn offers the patients more appointments to choose from. Finally, it simplifies course organization.

But here, too, the question arises, whether this affects the dentist-patient relationship and patient compliance. Patient compliance and motivation mediated by the person conducting the treatment are essential especially for the treatment of periodontitis. After all, continual plaque control is of the utmost importance and the essential first step in periodontal treatment according to the German S3 guideline [5–7]. To achieve sustainable improvement, patients must engage in time-consuming and sometimes extensive dental cleaning techniques [7].

In the student courses, the oral hygiene of patients is determined by standardized objective parameters within the framework of periodontal therapy. Short-term domestic plaque removal can be measured by the plaque control record (PCR) [8].

A low PCR means that patients make the best use of their abilities and show the best possible plaque reduction by cleaning their teeth as thoroughly as possible at home at least right before their treatment session [9, 10]. Long-term domestic plaque removal can be determined by the papilla bleeding index (PBI), which measures the extent of gingival inflammation [11–13]. Here, it should be taken in account that plaque-induced gingivitis only goes into remission after a few days of adequate plaque control [14]. In addition, there are parameters that broadly indicate the quality of periodontal treatment. Many studies propose reduced pocket depth, less movement in teeth, and raising the clinical attachment level as parameters for a successful periodontal therapy. The most valid and ideal parameter for predicting the further course of periodontitis is bleeding on probing (BOP) [13, 15, 16].

All these parameters allow measuring both the improvement of domestic oral hygiene and the quality of the treatment as objectively as possible. In dental student courses, it was noticed that periodontitis patients often showed inadequate oral hygiene in recall appointments. These cases had in common that the patients had so far been examined and treated by different students.

The view of oral microbiota and biofilms, which represent a key etiological factor for oral inflammatory diseases such as periodontitis, has become increasingly differentiated in the past decades. Recently, published reviews presented the oral biofilm as a highly dynamic environment for a variety of microorganisms, all of which interact with each other in a variety of ways and some of whose species differ considerably. There are some who even speak of a kind of acquired endogenous tissue layer. However, it is always the overall system of biofilm, host, and the treatment measures that determines the picture of disease or health [17–21]. Nevertheless, the most important measure for the prevention and treatment of periodontitis is still supra- and subgingival mechanical plaque control [7].

This randomized, controlled, prospective short-term study was conducted to determine whether treatment by different dental students affects domestic oral hygiene improvement and treatment outcome of patients in comparison to treatment by one single dental student, without losing sight of the overall therapeutic goal.

## Material and methods

### Study design

**Fig. 1:**
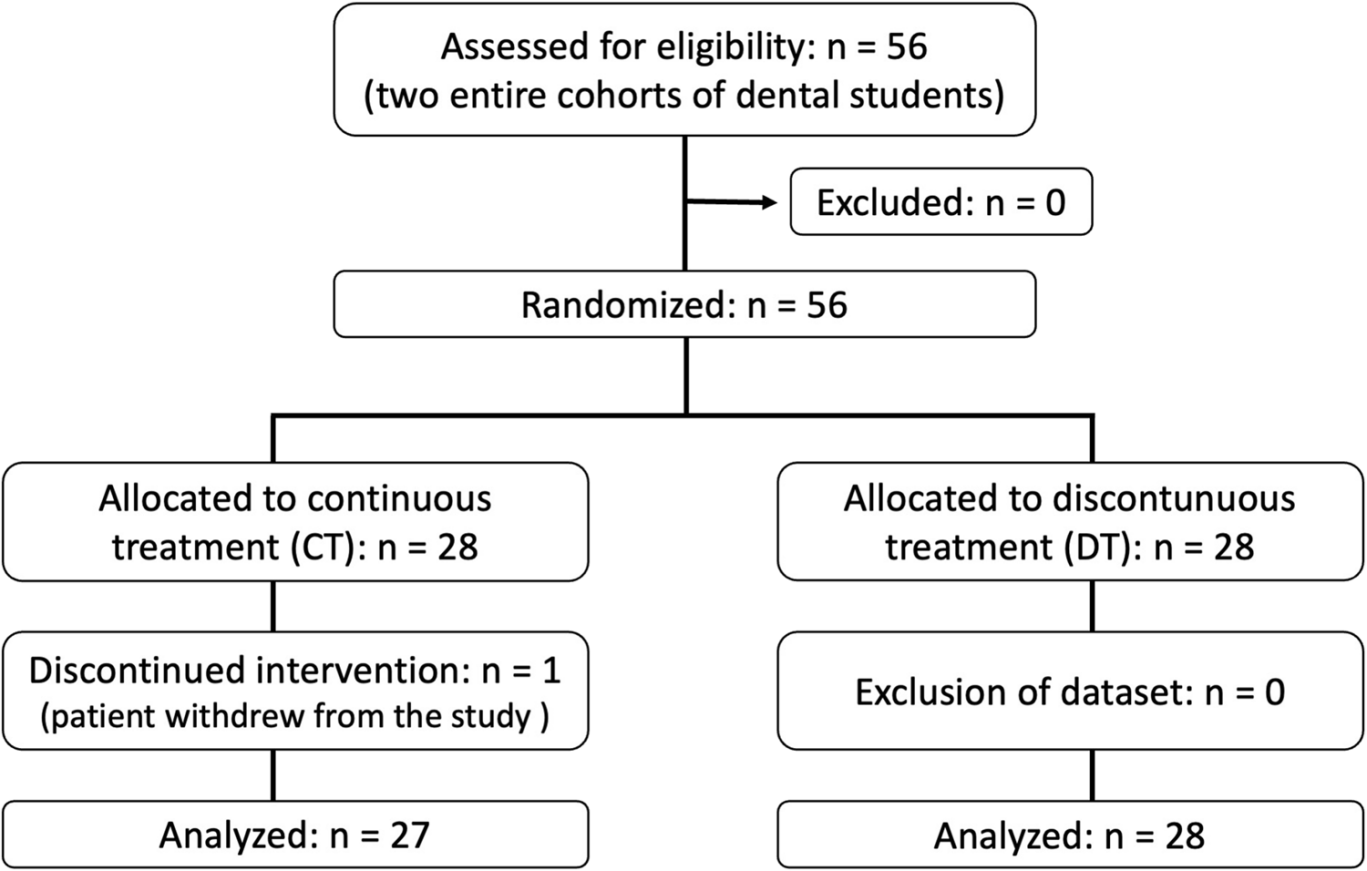
CONSORT flow diagram. The dental students were randomized into the treatment groups. Patients were allocated so that their prognostic factors were evenly distributed among the groups

This short-term study was designed as a prospective, randomized controlled trial, and approved by the local ethics committee (number 54/2011BO2). All participants gave written consent and could withdraw from the study at any time. Study participation did not result in any advantages or disadvantages concerning the performance of the dental students in the course. The study was performed in accordance with the Declaration of Helsinki (revised form, Seoul 2008), German Radiation Protection Ordinance, German Pharmaceutical Act (§§ 40-42), and the German Medicinal Devices Act (§§ 17-19). The study included 56 dental students randomized into two groups of equal size (Table 1). In the CT group (continuous treatment), the patients were treated by the same student in four consecutive treatment sessions. Each patient in the DT group (discontinuous treatment) was treated by a different student in each of the four treatment sessions. The treatment in both groups was identical. The dental students assessed the PCR, the PBI, and BOP at the beginning of the first, the second, and last treatment session. In this study, only the first and the last examination were evaluated. Dental students and patients were blinded to the study design and the study question (Fig. 1).

**Table 1:** Characteristics of participating dental students

Characteristics		All students	Students CT group	Students DT group
Gender	Female	28	12	16
	Male	27	15	12
Age (years)		26.5 ± 3.5	27.4 ± 3.9	25.6 ± 2.9

### Recruitment and randomization

#### Students

The participating students were all in the 4th year of the curriculum that comprises a total of 5 years according to the German licensing regulations [22]. The characteristics of the participating dental students are shown in Table 1. The allocation to group CT or group DT was performed by drawing lots.

#### Patients

Two entire cohorts from the dental school participated in the study. Patients with periodontitis were either recruited from the central patient admission unit or were referred from other departments. For the sake of comparability, a similar workload and the same treatment regimen was chosen for all students.

#### Inclusion criteria

- Agreement on treatment by dental students
- Pocket depth of at least 4 ml in combination with loss of interdental attachment on at least five teeth

#### Exclusion criteria

- Inability to perform oral hygiene procedures because of physical disabilities
- Infectious disease (e.g., HIV, hepatitis C)
- Life threatening disease (e.g., immunosuppression, leukaemia)

All patients were examined by a practitioner with periodontal experience prior to the study to characterize factors affecting the severity of periodontal disease (pocket depth). Furthermore, factors that can have an influence on oral hygiene and treatment response were recorded: smoking, diabetes mellitus, interleukin-1-polymorphism, and pathogenic microbial flora [23, 24].

Subsequently, the patients were manually allocated into two groups, i.e., continuous group (CT) and discontinuous group (DT) using a matched pair design to distribute the risk factors evenly among the groups (Table 2). Students and patients were blinded to the study question.

**Table 2:** Patient characteristics

Characteristics		All patients	CT group	DT group
			Continuous treatment	Discontinuous treatment
Gender	Female	22	11	11
	Male	33	16	17
Age (years)		54.9 ± 9.2	55.7 ± 9.9	54.0 ± 8.6
Smoking	Yes	25	14	11
	No	30	13	17
Diabetes mellitus		*n* = 6	*n* = 3	*n* = 3
Pocket depth	= 4–6 mm [%]	14.5	14.7	13.6
	> 6 mm [%]	5.0	5	4.7
Microbiological test	Type 4 or 5	*n* = 40	*n* = 17	*n* = 23
	Type 1, 2 or 3	*n* = 15	*n* = 10	*n* = 5
Interleukin-1 test	Risk A + B	*n* = 38	*n* = 16	*n* = 22
	Risk C + D	*n* = 17	*n* = 11	*n* = 6

*Pocket depth*:

• = 4-6 mm [%]: Average proportion of pockets in the group with a pocket depth between 4 and 6 mm

• > 6 mm [%]: Average proportion of pockets in the group with a pocket depth > 6 mm

*Microbiological test*:

• type 4 or 5: Number of patients whose microbiological test results correspond with type 4 or 5

• type 1, 2, or 3: Number of patients whose microbiological test results correspond with type 1, 2 or 3

*Interleukin-1 test*:

• risk A + B: Number of patients with normal reaction to inflammation or reduced anti-inflammatory effect

• risk C + D: Number of patients with strong or excessive inflammatory reaction and increased or highly increased genetic risk of infection

Allocation was done according to:

- Pocket depth (assessed with a pressure-controlled probe (Florida probe, Florida Probe Corporation, Gainesville, USA)).
- Smoking status (patients who had quit smoking more than 5 years earlier or had never smoked were categorized as non-smokers; patients who were currently smoking at least one cigarette per day or quit less than 5 years earlier were categorized as smokers).
- Any type of diabetes mellitus was recorded, yet the corresponding treatment was not.
- The interleukin-1-polymorphism was assessed with the GenoType®IL-1-Test (Hain Lifescience GmbH, Nehren, Germany). The test classifies the genetically determined production of interleukin-1 into the risk types A-D. A and B are normal levels of interleukin-1, while C and D denote excessive production and thus an enhanced inflammatory reaction.
- The pathogenic microbial oral flora was tested by the IAI-Pado-Test 4.5, Institute of Applied Immunology (Institut für angewandte Immunologie) — IAI, Zuchwil, Switzerland. The test determines the presence and combination of periodontal pathogens and classifies periodontitis therapy according to five statistically determined degrees of severity (types 1 to 5). The types 4 and 5 entail an unfavourable microbial composition, which necessitates antibiotic treatment in cases of severe periodontitis. Therefore, patients with types 4 and 5 were classified as high-risk for acquiring periodontitis, types 1 to 3 as low risk.

### Clinical procedures

**Fig. 2:**
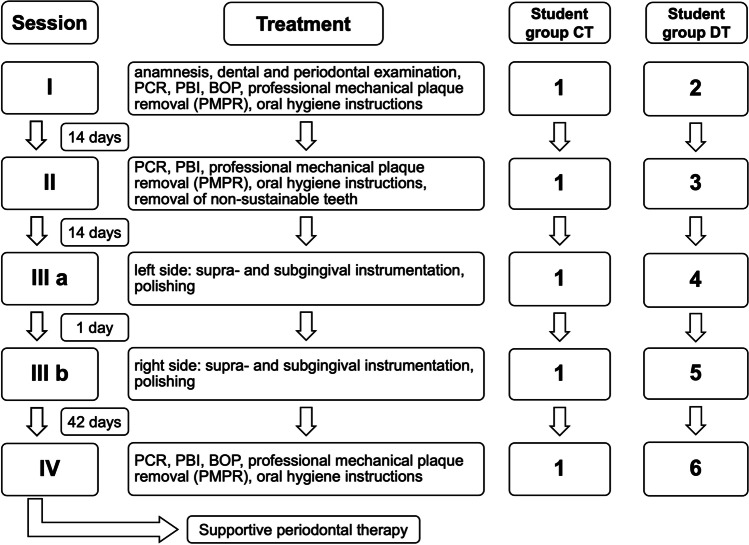
Treatment sessions and time intervals between the treatment sessions

The contents of the individual appointments were the same for all participants and took place at the same intervals (Fig. 2).

#### First treatment session

The students scoring the periodontal indices (PCR and PBI) provided standardized, yet individualized oral hygiene instructions, performed complete supragingival plaque and calculus removal, and concluded with a final polish of all teeth.

For domestic oral hygiene, it was recommended using the following:

- An electric toothbrush with oscillating and rotating cleaning action (e.g., Oral B, Procter & Gamble, Cincinnati, Ohio, USA).
- A less abrasive toothpaste, to minimize subsequent tooth sensitivity.
- A tongue cleaner with brushes or soft spikes to improve cleaning the tongue surface (e.g., OneDropOnly, Berlin, Germany).
- Individual interdental brushes adapted to the size of interdental spaces.

All patients acquired the requested cleaning utensils (electric toothbrush, less abrasive toothpaste, and tongue cleaner) prior to the first appointment.

The periodontal findings were comprised of probing depth, gingival recession, tooth mobility, the degree of furcation involvement, and BOP.

#### Oral hygiene instructions

During oral hygiene instructions, the students explained and demonstrated the correct use of both the electric toothbrush and the interdental brushes. The patients were instructed to use the electric toothbrush at least twice a day — in the morning and evening — for at least 2 min. It was recommended using the electric toothbrush in quadrants from posterior to anterior, focusing on each tooth by horizontal swivelling along the lateral surfaces and finishing by cleaning the chewing surfaces.

The training included selecting the correct size of interdental brushes for the different interdental spaces and bending the brushes to achieve access from buccal or labial to the interproximal spaces. The correct size was found, when the interdental brushes were gliding through the interdental spaces with slight pressure and a minimum of resistance. For interdental spaces where even the smallest brush could not fit, the patients were asked to use dental floss. A demonstration was given on how to pull the floss taut between two fingers and move it up and down between the teeth in a gentle sawing motion. Patients then performed the cleaning themselves by the aid of a mirror, under supervision and with feedback from the dental students.

#### Second treatment session

The second treatment session was scheduled two weeks later, at which point the dental students reassessed the periodontal indices (PBI and PCR). All teeth deemed non-sustainable were extracted and if necessary, replaced by a temporary prosthesis.

Like in the first session, the students performed supragingival plaque and calculus removal and recapitulated all oral hygiene instructions. Sometimes a larger size interdental brush had to be used, since the inflamed swelling was somewhat reduced by the domestic hygiene measures.

#### Third treatment session

At the third treatment session, which took place another two weeks later, the dental students performed subgingival cleaning including scaling, root planning, and tooth polishing under local anaesthesia. When more than eight teeth required treatment, the third session was split into two appointments on two consecutive days. For scaling and root planning, the students used tooth area specific Gracey curettes (no. 1/2, no. 7/8, no. 13/14, no. 15/16, Hu-Friedy Mfg. Co., 3232 N. Rockwell St., Chicago, IL). The pockets were irrigated with 0.2% chlorhexidine solution.

#### Fourth treatment session

The fourth treatment session took place 42 days after the third treatment session.

The students once again reassessed the periodontal indices PCR, PBI, and BOP and again performed professional supragingival plaque removal, as well as subgingival scaling and root planning of residual pathological periodontal pockets.

Consequently, the total duration of active periodontal treatment was 70 to 71 days.

### Periodontal indices and examinations

During the first, second, and fourth treatment session, the dental students evaluated three oral hygiene and periodontal indices. Only the first and last examination was evaluated in this study. To assure the accuracy of the data, all students were instructed both in theory and practice on how to assess the data and how to use standardized forms for documentation. The accuracy of the data was monitored by an experienced periodontist. The dental students assessed only those teeth considered worth saving.

#### Papilla bleeding index

The PBI was assessed according to the original publication by Saxer and Mühlemann [12]. The dental students used a standardized periodontal probe (WHO probe) for gently exploring the gingival sulcus towards the interproximal dental papillae of each quadrant alternating between the buccal and oral side.

The PBI, however, includes not only the occurrence of bleeding in the interdental spaces but also the intensity of bleeding (Table 3).

**Table 3:** Papilla bleeding index for scoring

0	No bleeding
1	One bleeding point
2	Several bleeding points or one thin bleeding line
3	Interdental triangle filled with blood
4	Profuse bleeding, blood spreads towards the marginal gingiva

**Fig. 3:**
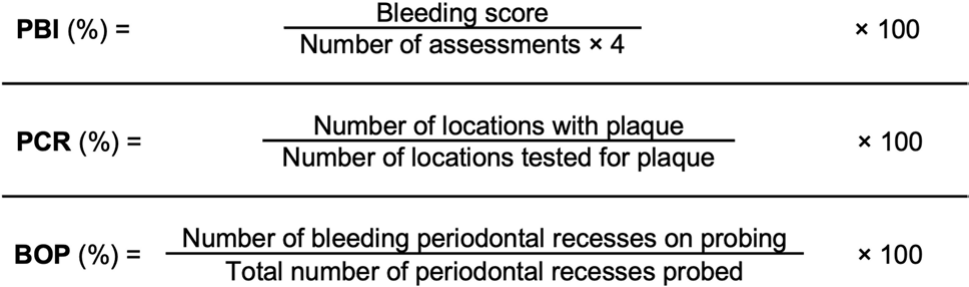
Formulas for calculating PBI, PCR, and BOP

In case of bleeding, the intensity was documented on a standardized form. The sum of all recorded bleeding spots gives the bleeding score [25]. The PBI was calculated according to the formula in Fig. 3.

#### Plaque control record

The surface of each tooth was swabbed with a red plaque indicator (Rondell red, Fa. Directa, Stockholm, Sweden). After rinsing the mouth, the dental students recorded on a standardized form, whether there was plaque either on the mesial, buccal, distal, or oral surface. The PCR was calculated according to Fig. 3.

#### Bleeding on probing

**Fig. 4:**
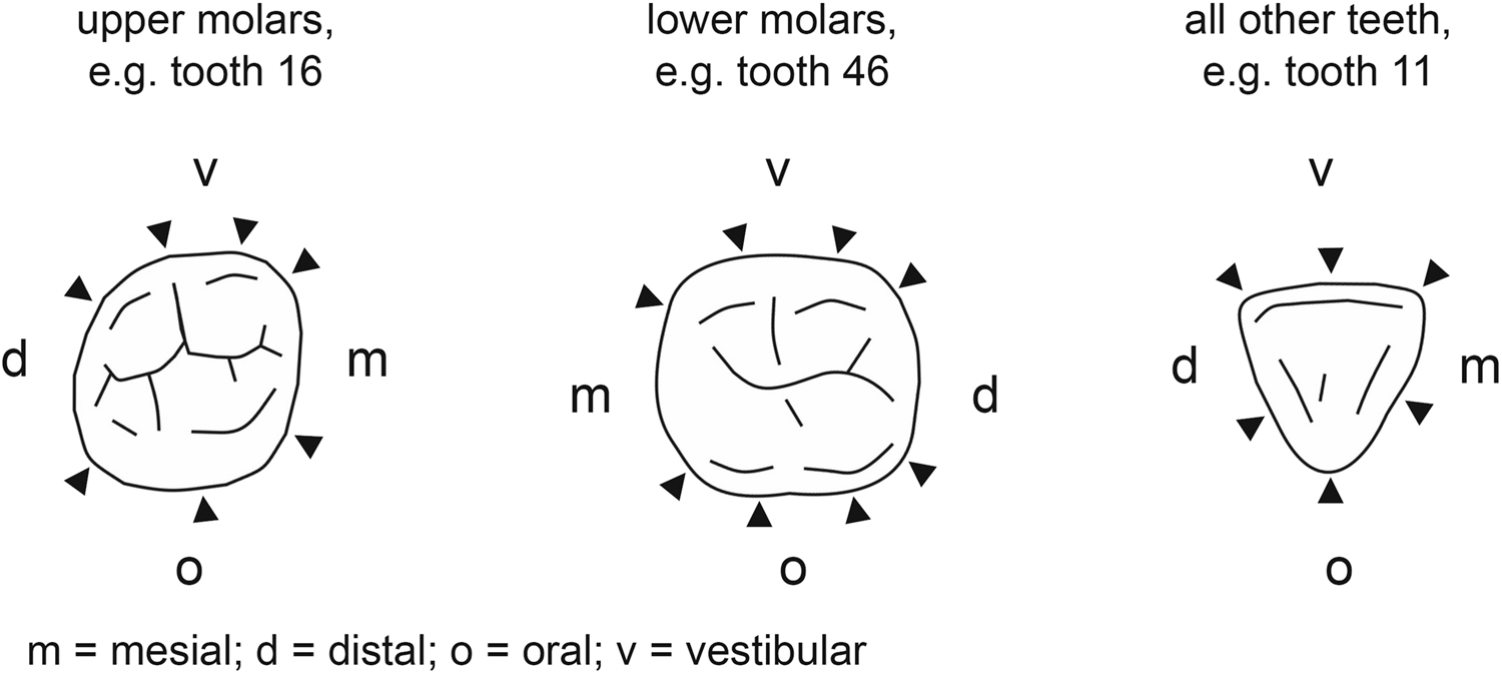
Triangles representing the probing locations for assessing BOP

The upper molars were gently probed to pocket depth at seven spots, the lower molars were probed at eight spots, and all other teeth at six with the blunt end of the periodontal probe (Fig. 4). The dental students recorded, whether bleeding was visible within 30 s. The results were documented on a standardized form. BOP was calculated according to Fig. 3.

### Statistical analyses

Statistical analyses were performed by JMP 16 software (SAS Institute GmbH, Heidelberg, Germany).

The results were analysed in three different ways. For both the DT and the CT group, intragroup as well as intergroup comparison of the values of BOP, PCR, and PBI was performed at the beginning (session I, T1) and at the end (session IV, T2). All values were assessed according to the Gaussian distribution before tests were performed. A *p*-value < 0.05 was considered statistically significant. Due to a lack of normal distribution of the values (Anderson darling test: *p* < 0.05), the data were screened for statistical significance using the non-parametric Wilcoxon tests. The categorization of patients in terms of clinical parameter improvement was based on the ∆ (T1-T2) of the clinical parameters BOP, PCR, and PBI. All values > 0 were categorized as improvement. Based on these results, a contingency analysis was performed with the two-sided chi-square test. Spearman’s rank analysis was performed to observe possible correlations between the clinical parameters and the age of the patient. Due to the nature of the study as an explorative trial, no adjustment of *p*-values was done.

## Results

In total, 55 patients (22 females and 33 males, mean age 54.9 ± 9.2 years) completed the study. One participant dropped out due to hospitalization for several weeks in need for major surgery, unrelated to the dental treatment.

Twenty-five patients were smokers (group *DT* = 11, group *CT* = 14), six patients suffered from diabetes mellitus (3 patients in each group). The average proportion of pockets with a pocket depth between 4 and 6 mm was 14.5% (*DT* = 13.6%, *CT* = 14.7%), the average proportion of pockets > 6 mm was 5% (*DT* = 4.7%, *CT* = 5%) (Table 2).

There was neither an impact of the microbial biotype nor of the genetic risk factor on the change of the clinical parameters from T1 to T2 (data not shown).

**Fig. 5:**
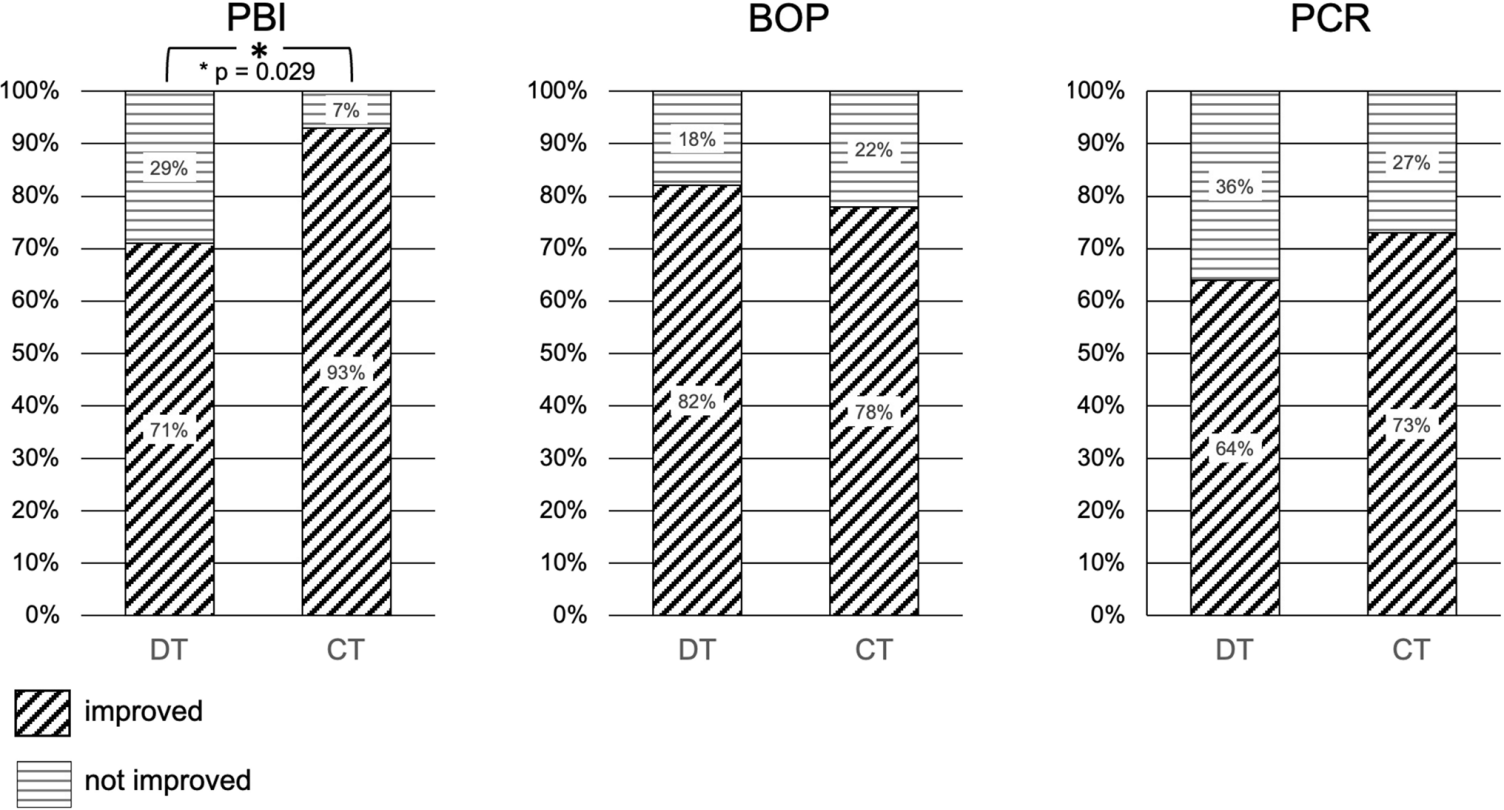
Papilla bleeding index (PBI), bleeding on probing (BOP) and plaque control record (PCR), and the relative number of patients that showed improvement or non-improvement in group DT (discontinuous treatment) and CT (continuous treatment)

  In the CT group, 93% of the patients showed an improvement of PBI compared to 71% in the DT group (Fig. 5). This difference was statistically significant (*p* = 0.029). The corresponding median ∆ PBI was 12.5 (*IQR* 3.57-22.2) for CT and therefore more pronounced compared to 6.08 ( -1.18-15.95) for DT, in any case with no statistical significance (Table 4). Regarding the parameters BOP and PCR, the relative number of patients with improvement was almost equal in comparison of both groups (Fig. 5). Similarly, the ∆ BOP and ∆ PBI displayed comparable values (Table 4).

**Table 4:** Quantile-scores (in %), inter-group ∆, and intra-group *p*-values of the DT group (discontinuous treatment) and the CT group (continuous treatment)

Parametre	Group	∆ T1-T2			Session I			Session IV			∆ Inter-group	Intra-group *p*-value
		%			%			%				
		median	25	75	median	25	75	median	25	75		
BOP	DT	** 8.90**	3.33	16.56	**34.79**	23.03	47.90	**25.55**	11.88	33.10	0.919	< 0.001
	CT	** 9.40**	2.16	20.71	**29.80**	19.61	62.30	**17.00**	6.40	36.90		< 0.001
PCR	DT	**19.50**	6.28	46.03	**66.15**	58.35	80.03	**41.30**	25.67	58.60	0.333	< 0.001
	CT	**16.66**	5.55	26.1	**69.59**	45.23	78.37	**47.73**	22.50	62.50		< 0.001
PBI	DT	** 6.08**	-1.18	15.95	**15.48**	6.87	34.77	** 9.40**	4.56	20.56	0.117	0.027
	CT	**12.50**	3.57	22.2	**29.38**	13.22	44.19	**10.57**	2.90	28.33		< 0.001

Both groups showed a statistically significant improvement of all observed clinical parameters from T1 to T2. The comparison of the median ∆ T1-T2 between the groups was most pronounced for the PBI in the CT group (T1: 29.38 (13.22-44.19), T2: 10.57 (2.90-28.33) (Table 4).

 Correlation analysis of age, BOP, PBI, and PCR revealed a weak correlation between ∆ PBI and ∆ BOP as well as for ∆ PCR and ∆ BOP (Spearman’s *ρ*_*s*_ = 0.387 and 0.244, resp.), showing a statistically significance for ∆ PBI/∆ BOP). Analysing each group showed comparable results for the CT group, whereas the correlations were less pronounced within the DT group (Table 5).

**Table 5:** Correlation analysis of age, BOP, PBI, and PCR for the DT group and the CT group

Variable	With variable	Group DT		Group CT	
		Spearman *ρ*	*p*-value	Spearman *ρ*	*p*-value
∆ BOP	Age	-0.1136	0.5649	0.1939	0.3325
∆ PCR	Age	-0.0233	0.9064	-0.0606	0.7642
∆ PCR	∆ BOP	0.1648	0.4021	0.2766	0.1626
∆ PBI	Age	-0.0762	0.7000	-0.1234	0.5397
∆ PBI	∆ BOP	0.1716	0.3825	0.5853	0.0013
∆ PBI	∆ PCR	0.1084	0.5830	0.2183	0.2740

## Discussion

Treatment in dental medical care centres as well as in university dental clinics might often be carried out by different practitioners. As there might be a conflict between the goals of dental schools to teach their students on as many different patients as possible on the one hand and to give them the opportunity to establish a patient-dentist relationship to facilitate oral hygiene compliance on the other hand, the present study investigated whether treatment by several different dental students compared to treatment by a single student influences the oral hygiene of patients.

In dental care centres, instructions and therapy methods may vary from operator to operator, while in this study, oral hygiene instructions were given according to a standardized protocol. The growing number of dental care centres throughout Germany entails that a change of practitioner during a therapy period could be a phenomenon that occurs more often in the future [1]. Moreover, it can be assumed that due to a heterogenous employee structure within medical care centres, the different dental practitioners obtained their degrees from different universities in the country and abroad and gained their skills and knowledge from different educational facilities. Thus, it is inevitable that a variety of acquired therapeutic concepts and approaches will be applied. Therefore, it could be hypothesized that changing practitioners within dental care centres might affect treatment results even more than changing practitioners with a standardized protocol.

Establishing good oral hygiene over a long period of time is one of the most important prerequisites for treating periodontitis [26].

This study showed that both indices relating to oral hygiene (PCR and PBI) as well as the bleeding tendency of the periodontal pockets (BOP) within the groups both when treated by the same practitioner (CT group) and by different practitioners (DT group) had improved at the end of the observation period.

One reason for this could be that any kind of professional intervention, e.g., two supragingival professional tooth cleanings at different times or scaling and root planning, has an influence on the patient’s oral hygiene, regardless of the number of practitioners. This finding is not new. As early as the 1970s, numerous studies showed that regular professional tooth cleaning or interventions in domestic oral hygiene have a beneficial effect on the oral hygiene status of patients and as a result plaque and gingival index scores improve [27–30].

Until now, many patients used only manual toothbrushes, and the few who used dental floss did so only occasionally. The transition to the recommended aids has certainly contributed to this improvement, although it is impossible to verify that every patient has used the aids regularly and as instructed. Several studies and meta-analyses in recent years have shown that the use of the oral hygiene aids recommended in this study generally leads to an improvement of the gingival situation [31–36].

The PBI improved statistically significantly more frequently in the CT group compared to the DT group. In contrast, BOP and PCR improved in an equal number of patients comparing CT and DT. For the BOP, this means that the inflammation of the pockets has improved significantly most likely due to the subgingival instrumentation. Changing practitioners should not influence this parameter, as it is less dependent on the patient’s behaviour than on the correctly performed subgingival debridement [14].

PCR is a parameter that patients can easily influence by brushing their teeth very intensively immediately before the dental appointment and thus actually generate false-positive values regarding the quality of domestic oral hygiene. Therefore, it is interesting that the improvement in the PBI was seen in more patients of the CT group compared to the DT group. This could indicate that a constant practitioner might have stronger influence on the tooth cleaning compliance compared to changing practitioners from session to session. It would also be in agreement with the findings of previous investigations: In a study by Stacey in 1978, the treatment success depended more on the relationship between dental student and patient than on socio-economic factors [37]. In 2014, Jones et al. found empathic communication superior to merely informing the patient about dental procedures where treatment adherence is concerned. It might be assumed that forming a stable relationship between dentist and patient is a prerequisite for empathic communication [38]. However, in 2012, Sachdeo et al. compared patient complaints about primary care settings versus dental schools, and discontinuity of treatment was not one of the most important concerns of the patients [39].

In addition, this short-term study analysed possible correlations between the clinical parameters. Interestingly, the correlations between ∆ PCR and ∆ BOP as well as ∆ PBI were more pronounced in CT compared to DT. This might indicate that the domestic plaque control activities in the CT group affected the inflammatory parameters more distinctly than in the DT group. Furthermore, the correlation between ∆ PBI and ∆ BOP was significantly higher in the CT than in the DT group. Again, considering that ∆ BOP is more influenced by the dental treatment from practitioners and the PBI is more influenced by the patient’s domestic hygiene [40–42], this could be interpreted that the stronger correlation in the CT group indicates both successful treatment by the practitioner and at the same time more pronounced patient compliance.

Only one patient from the continuous group withdrew from the study due to hospitalization for treatment of a severe condition unrelated to the dental treatment.

Therefore, one might speculate that patients and dental students are able to establish a trusting relationship that promotes compliance as long as there is continuity in the treatment of patients by the same treatment provider. Indeed, questioned about their opinions regarding periodontal treatment, dental students in Colorado mentioned that the treatment by multiple providers interfered with providing good periodontal treatment [3]. Butters and Willis and Ebn Ahmady et al. reported high overall patient satisfaction with treatment provided by dental students [43, 44].

However, none of the investigators directly compared the effect of continuous versus discontinuous treatment on patient satisfaction or treatment success. Furthermore, no studies could be found that compared the treatment outcomes of patients, who were either treated by consecutively different practitioners for each treatment step or had the same practitioner for the entire treatment.

Recently, a debate has arisen about whether motivational interviewing or participatory goal setting should be included as components of therapy, as for instance mentioned in the new diabetes type 2 guideline, and whether this might have a compensatory effect on treatment by several practitioners [45]. The correct application of such accompanying measures naturally also requires time and training. Nevertheless, it could perhaps at least somewhat reduce the deficits of domestic oral hygiene. In general, communication and structured conversation techniques must also be discussed as important components of the dental curriculum in the future.

The main reason patients came to the clinic was them suffering from periodontal disease. For this reason, the most important factors with an influence on periodontitis treatment were determined right away, in particular smoking and diabetes mellitus, to be able to assign the patients to both groups in the best possible way in a matched-pair design [23, 24]. The significance of the interleukin I polymorphism and microbiological analysis has meanwhile been viewed very critically by some reviews and meta-analysis [46–50]. Nevertheless, both tests were used in this study to facilitate the detection of patients with possibly existing altered inflammatory processes or a significantly changed pathogenic germ flora and to distribute these patients between the two groups.

Particularly in recent times, numerous review papers have been published addressing the development and maintenance of gingivitis and periodontitis with particular reference to the oral microbiome of the host. The oral microbiome represents a highly dynamic environment for a variety of microorganisms, all of which interact with each other in a variety of ways and whose species differ significantly in the progression from gingivitis to periodontitis [17, 21]. However, it must not be forgotten that it is always the entire system of microbes, host, and the treatment measures or their joint interaction that determine the picture of the disease, but of course also of the state of health [20].

Thus, in addition to the acquisition of external microbes, the host’s diet, salivary flow, and even chewing forces have an influence on this system.

For Kaan et al., it is important to note that the oral microflora in conjunction with the host represents a dynamic process that is subject to very different degrees of expression and, depending on the acquired microbial species, interactions in the individual life stages, from the fetus to puberty [19]. Its composition is, amongst other things, determined by factors such as sugar intake, oral hygiene habits, antibiotic use, smoking, and social factors.

Darveau et al. go even further and describe the oral biofilm as a tissue-mimicking entity, as it were a kind of acquired endogenous tissue layer with specific, also innate, functional properties, which can lead to excessive inflammation and tissue destruction when disturbed, for example by new, previously non-local bacteria [18].

With this in mind, the results of this study should be interpreted to mean that although domestic oral hygiene has a major influence on the degree of inflammation of the gingiva and periodontal structures, it is not the only factor. However, these more recent findings, particularly in the area of nutrition, were not included in the study and had not yet been incorporated into the curriculum at the time the study was conducted. Nonetheless, these studies show that these findings must also be incorporated into dental education and considered in future studies, including long-term studies, and discussed with the patient as part of the treatment plan.

The present study has some limiting factors. The study period is very short due to the chosen setting within an educational curriculum. The curriculum structure does not allow a longer period of time that would be required to draw randomly transferable conclusions. Nevertheless, even with longer study periods in the context of supportive periodontal care, the results may point towards differences between one practitioner as a permanent health care professional and constantly changing practitioners. However, in the future, the new study regulations in Germany might open up new opportunities to supervise and conduct similar studies on a prolonged basis. As there are very few short- and long-term randomized controlled studies to date, future studies should be conducted to examine the effect of changing practitioners over longer observation periods, including SPC, and beyond to other behaviour change topics that are likely to require a sufficient practitioner-patient relationship, such as nutrition counselling.

Furthermore, it is a single centre study with a rather small-sized study sample. Nevertheless, because two whole cohorts of students were able to be recruited, it may be assumed that the sample is representative in terms of dental students. The patients were allocated manually with a matched-pair design to avoid imbalance in disease severity or in other factors affecting the success of treatment. While the investigator balancing the study sample was not blinded to the study question, he had only access to the patient files. This way, there were no actual clues allowing the prediction of future compliance, and after sorting the patients into two groups, the groups were randomly assigned to the intervention procedures.

In smokers, the BOP may be unreliable; so far, studies have either found no effect or a strong suppressive effect of smoking on gingival bleeding because of the reduced blood perfusion of gingival tissues [51, 52]. As expected, in this study, smokers were less likely to improve in BOP, and thus achieve non-inflamed pockets, than non-smokers. Where the other indices are concerned, smokers had an even better PCR and PBI than non-smokers (data not shown). Even though smokers were slightly overrepresented in the continuous group (14/27 versus 11/28 in the discontinuous group), it is assumed that this did not distort the results in a crucial way.

The comparison between groups at the start of therapy showed a heterogeneous starting position in relation to the parameters for this study. If the BOP value in the DT group was higher than in the CT group, it was the other way around for PCR and PBI.

Basically, the initial determination of PBI, BOP, and PCR could have been performed in session I, to possibly distribute patients more evenly based on their parameter values. However, this would have made course planning much more difficult and possibly also resulted in limitations, such as an unfair distribution of the periodontal pockets to be treated per student, difficulty in assigning the treating student (CT or DT), and, of course, an uneven distribution of risk factors. In this context, Tonnetti et al. demonstrated that patients who received thorough subgingival cleaning of the periodontium had both lower plaque scores and lower rates of gingival bleeding two and  six months after periodontal therapy than patients in the control group who received only supragingival cleaning [53]. This could introduce a slight bias in the results, although in the present study subgingival cleaning was performed in both groups.

Periodontitis is a chronic disease and successful treatment requires sustained effort on part of the patient and the dentist. Also, only the treatment phase was investigated, not the preservation phase. Hence, this study covered only a short period of time. It is reasonable to suspect that oral hygiene, as already mentioned in the introduction, deteriorates even further if the time between recall appointments becomes longer.

## Conclusions and recommendations

Although there were no differences regarding the change of clinical parameters between the groups, we found a significant higher number of patients with PBI improvement when they were treated by one single practitioner. Therefore, the present analysis suggests that further research should investigate treatment by one and the same practitioner or every time a different one over a longer observation period. Based on our data, courses for dental students should possibly be planned in a way to enable continuous treatment of each patient by the same student. In the future, the new German study regulations might open up new opportunities to supervise and conduct similar studies on a prolonged basis.

Similarly, at dental medical care centres, long-term therapies or treatments that require ongoing follow-up appointments might be better performed by the same dentist or prophylaxis staff.
